# Sadism and Personality Disorders

**DOI:** 10.1007/s11920-023-01466-0

**Published:** 2023-10-19

**Authors:** Jill Lobbestael, Ghizlane Slaoui, Mario Gollwitzer

**Affiliations:** 1 Clinical Psychological Science, Faculty of Psychology and Neuroscience, Maastricht University, Maastricht, the Netherlands; 2 Faculty of Psychology and Neuroscience, Maastricht University, Universiteitssingel 40, Maastricht, 6229 ER the Netherlands; 3 Department of Psychology, Ludwig-Maximilians-Universität, Munich, Germany

**Keywords:** Sadism, Sadistic pleasure, Personality disorders, Personality, Dark Triad

## Abstract

**Purpose of Review:**

Sadistic pleasure—the enjoyment of harm-infliction to others—can have devastating interpersonal and societal consequences. The goal of the current review is to illuminate the nomological net of traits related to sadism. We aim to achieve an understanding of the current empirical status on the link between sadism and personality disorders, psychopathy, the Dark Triad, and basic personality traits in clinical and community-based samples.

**Recent Findings:**

The field is dominated by self-report studies on the Dark Triad with convenience samples. The link with DSM personality disorders has hardly been empirically studied. Existing evidence shows that sadism is most strongly related to increased psychopathic personality traits.

**Summary:**

Sadism can originate both from the interpersonal, affective, and behavioural basis of dark personality traits. There are diverging ideas on the differential status between sadism, psychopathy, and other dark traits. Research is needed on the causal impact of the broader range of personality disorders on sadism, in more diverse samples, including behavioural assessments of sadistic pleasure, as well as on the interplay of such personality traits with situational and affective aspects, and victim attitudes.

## Introduction

Sadism refers to the pleasure that can arise from the physical or emotional suffering of others [1–4]. Sadism spans from enjoying seeing others in pain not inflicted by oneself (i.e. indirect/vicarious sadism) to actively engaging in sadistic behaviours (i.e. direct sadism), where one is responsible for other people’s pain [5]. The vast majority of sadism research focusses on sexual sadism, with an estimated 2–5% of people obtaining sexual gratification from inflicting pain on others [6–8]. This might have contributed to the common belief that sadism is a rather rare phenomenon. Nonetheless, some authors refer to the presence of ‘everyday sadism’, which implies ‘largely acceptable forms of subclinical sadism that are prevalent in modern culture’ [9], as reflected by the popularity of violent movies, video games, and brutal sports [10, 11]. Such subclinical sadism has been reported by around 7% of student samples [12], although actual prevalence rates may be higher because of social desirability biases in self-report measures. Some authors even claim that most individuals have the capacity to indulge in the rush of pleasure that is evoked by sadistic behaviour [10].

There has been a continuous debate about the diagnostic status of sadism. In the late 1980s, sadistic personality disorder appeared in the appendix of the DSM-III-R [13] as a personality disorder to be considered for future DSM editions. The core feature of sadistic personality disorder was a pervasive pattern of ‘cruel, demeaning, and aggressive behaviour, for the purpose of amusement or obtaining pleasure from the suffering of others’ [13, p. 371]. However, sadism was not included as a personality disorder diagnosis in later DSM versions because it was considered insufficiently distinct from antisocial and narcissistic personality disorders to warrant a stand-alone disorder [14, 15]. By contrast, sexual sadism disorder was always a separate disorder in the DSM editions, albeit under different names. In the current DSM-5 version [16], it is part of the algolagnic disorders—a class of paraphilic disorders wherein sexual arousal is dependent on pain and suffering.

As a consequence of the historical changes in sadism’s position as an official diagnosis, its operationalization differs widely across empirical studies. In violent offender samples, case files are often coded based on characteristics of sadistic criminal acts (e.g. torture or recording crimes or sexual offenses aimed at deriving sexual pleasure from hurting others). By contrast, self-report measures of sadism do not only measure sadistic behaviour, but also sadistic thoughts or fantasies. Sadism studies rely almost exclusively on self-report measures, operationalized as a dimensional personality trait. Examples of such measures are the *Assessment of Sadistic Personality* (ASP; e.g. ‘I never get tired of pushing people around’) [17], the *Short Sadistic Impulse Scale* (SSIS; e.g. ‘I enjoy seeing people hurt’) [18], and the *Varieties of Sadistic Tendencies Scale* (VSTS; e.g. ‘I enjoy hurting people’, ‘I dominate others using fear’) [11, 19]. The measurement properties of these scales differ, but they do predict sadism-related behaviour in the lab (e.g. antisocial punishment [20]; aversive noise blasts administered to an alleged opponent [21]) and online (e.g. trolling behaviour [22]). Although ethical and practical constraints make it difficult to study sadism in the laboratory, some researchers made use of a simulated bug-killing procedure developed by Martens and colleagues [23••] to test at a behavioural level whether people aggress and derive pleasure from doing so.

Sadism can have devastating interpersonal and societal consequences. Because of its highly destructive nature, it is important to understand interpersonal and context variables that heighten the risk of sadism. The current review aims to illuminate the nomological net of traits related to sadism. We will summarize the literature of the past 3 years on the link between sadism and personality disorders (traits) in clinical and community-based samples. Because of the scarce literature on sadism and the 10 distinct DSM personality disorders, we will also turn to literature on psychopathy, the Dark Triad, and basic personality traits such as the Big Five.

## Literature Review: Sadism and Personality Disorders

### Sadism and Personality (Disorders) in Clinical Samples

The main personality constellation that has been linked to sadism is psychopathy, which is characterized by deficits in emotional functioning and antisocial behaviour, such as superficial charm, irresponsibility, fearlessness, conning behaviour/manipulation, and lack of empathy [24]. Psychopathy is not listed as an official clinical personality disorder diagnosis in the DSM-5 or the ICD-11 that instead classify psychopathy as an antisocial personality disorder (ASPD, [16]) and dissocial personality disorder (DPD, [25]), respectively. The factor structure of psychopathy has been subject to debate; for a long time, researchers accepted a two-factor model of psychopathy that consists of core interpersonal–affective personality traits (factor 1) and impulsivity–antisocial behaviour (factor 2) [26]. Later, four-factor models were suggested implying that psychopathy consists of four correlated dimensions that reflect specific interpersonal, affective, lifestyle, and antisocial features [27]. The most widely studied clinical sample in the context of sadism is male forensic patients. A recent meta-analysis over 19 samples with 5161 patients found medium-size associations between total psychopathy interview scores and sadism (*r* = 0.24) [28••]. The links between the two psychopathy subfactors and sadism were of comparable strength (*r* = 0.25 and *r* = 0.26, for factor 1 and for factor 2, respectively). Although additional analyses indicated that the sadism-psychopathy link was unrelated to the assessment method of sadism or to the type of sadism (i.e. direct versus indirect), the meta-analysis was likely underpowered to conclude this as almost all studies used clinician-rated sexual sadism disorder or sadistic elements of the perpetrated acts (e.g. torture of recording crimes [28••]) as sadism measures. A recent study relying on self-reported sadism in convicted male prisoners confirmed that all psychopathic components (i.e. interpersonal, affective, lifestyle, and antisocial) are related to increased sadism [29]. In another male sexual homicide offender sample, psychopathy factor 1 was more strongly related to sexual sadism in crime scene behaviour, while factor 2 related more strongly to a DSM-IV sadism disorder [30].

Aside from psychopathy, the link between sadism and other personality disorders has barely been studied. One exception is a study among male sexual homicide offenders, where the level of sexual sadism in crime scene behaviour showed to be positively associated with a clinical diagnosis of obsessive–compulsive personality disorder [30]. In another study, narcissistic personality disorder patients reported higher levels of sadism compared to a mixed group of borderline, dependent, avoidant, and passive-aggressive personality disorder patients [31•].

### Sadism and Personality (Disorders) in Community Samples

The vast majority of studies on sadism and personality in community samples focus on Dark Triad traits. The Dark Triad is composed of three socially maladaptive personality traits: narcissism, Machiavellianism, and psychopathy [32]. All three concepts are underpinned by shared callousness, self-promotion, and social deviance [32, 33]. Machiavellianism and psychopathy further share self-control deficits [34]. In addition, narcissism implies a pattern of attention-seeking and fantasizing about unlimited success or power and possessing a grandiose sense of importance and entitlement [35]. Machiavellianism refers to deception, manipulation, and a common use of deceit, flattery, or cynicism to promote one’s own interests [36]. All Dark Triad traits have been linked to increased levels of self-reported sadism. Two recent meta-analyses concluded that among the Dark Triad traits, psychopathy had the strongest link with self-reported sadism (*r*’s = 0.58; whereas *r*’s = 0.26–0.27 for narcissism; and *r*’s = 0.43–0.46 for Machiavellianism [37••, 38••]. Two large studies with undergraduate student samples differentiated between the different types of sadism and showed that both direct and vicarious sadism related to increased levels of all Dark Triad traits [39•, 40•], except for narcissism and vicarious sadism in one of these studies [39•]. An online study showed that psychopathy was the only Dark Triad component that predisposed to sexual sadism [38••].

Another line of research focused on sadism and subclinical psychopathy outside the context of the Dark Triad. Overall, depending on the specific questionnaires that were used, psychopathy and sadism were correlated between *r* = 0.21 and 0.78 [41•, 42•]. A large community-based study differentiated between primary (callous, manipulative, selfish, routinely untruthful) and secondary (emotional dysregulation, impulsivity, and social dysfunctioning) psychopathy and found that both types are related to direct as well as vicarious sadism [43•]. Other studies aligned with psychopathy’s triarchic model [44], which has been supported using non-clinical samples, relying on assessment instruments that omit criminal and antisocial behaviour [45]. These subclinical psychopathy factors have been labelled fearless dominance, self-centred impulsivity, and coldheartedness [46]. Two recent studies on the triarchic psychopathic model found that self-centred impulsivity is related to increased self-reported sadistic tendencies in both male [47•] and predominantly female community samples [48•], while the coldheartedness factor was linked to increased reported sadism in the latter sample only [48•]. Two recent behavioural studies showed that psychopathy’s coldheartedness factor is related to increased pleasure after exerting sadistic behaviour (i.e. ostensibly grinding bugs) in the lab [47•, 48•].

Research on sadism and DSM personality disorder traits in community samples is scarce. One large-scale online study showed that both direct and vicarious sadism are related to increased vulnerable narcissistic and borderline personality disorder traits [43•]. In an unpublished study from our lab [49], *N* = 120 community participants completed the Assessment of DSM-IV Personality Disorders questionnaire (ADP-V) [50]. We found that cluster-B traits positively predicted self-reported sadism (total sadism: *β* = 0.65; verbal: *β* = 0.52; physical: *β* = 0.54; vicarious: *β* = 0.35; *p*s < 0.001^1^), while cluster-C traits emerged as a negative predictor (total sadism: *β* =  − 0.22; verbal: *β* =  − 0.24; *p*s = 0.02). When the four separate cluster-B disorders where entered as predictors, antisocial personality disorder showed to relate to all forms of sadism (total sadism: *β* = 0.61; verbal: *β* = 0.26; physical: *β* = 0.38; vicarious: *β* = 0.49; all *p*s < 0.001), while narcissistic traits related to increased direct sadism (verbal: *β* = 0.24; physical: *β* = 0.26; *p*s < 0.001)*.*

One final line of relevant research focusses on the link between sadism and the basic personality factors in the Big Five and HEXACO models [51, 52]. Questionnaire studies found that sadism was negatively correlated with agreeableness [39•, 40•, 53], honesty-humility [39•, 40•, 53, 54], conscientiousness [39•, 40•, 53], emotionality [39•, 40•, 53], and openness [53]—painting a picture of sadistic individuals as lacking integrity, emotionality, discipline, kindness, and social activity.

## Discussion

### Overall Findings

Three main conclusions can be drawn from the present review. *First*, the link between sadism and the 10 DSM-defined personality disorders has hardly been studied. We counted only two clinical and two non-clinical studies. *Second*, the personality traits most often studied in relationship to sadism are psychopathy and narcissism, either as stand-alone or flanked with Machiavellianism in the context of the “Dark Triad”. This evidence stems both from forensic and community samples. Studies in community samples further show inverse relationships between sadism and more general (Big 5/HEXACO) personality factors. Sporadically, (sexual) sadism is linked to obsessive–compulsive and borderline personality disorder traits. *Third*, there is not much evidence for high specificity within the found sadism-personality links, as all psychopathic subfactors are related to increased sadism, and direct versus vicarious subforms of sadism do not appear to differentially relate to personality (disorder) traits.

### Behavioural Versus Personality Components

Sadism appeared to be most strongly related to dark and dramatic personality traits—as featured in antisocial, psychopathic, and narcissistic personalities. While some of these traits largely represent affective/interpersonal components, like grandiosity and lack of empathy (central to narcissism, and psychopathy’s fearless dominance and coldheartedness factors), others reflect a behaviourally deviant repertoire (central to psychopathy’s self-centred impulsivity, and most antisocial personality disorder traits). The current review suggests that sadism relates to both components. This indicates that sadism has an interpersonal/affective basis but is also related to behavioural patterns of impulsivity and rule-breaking.

This first finding implies that those who enjoy hurting others either have an over-exaggerated sense of self-importance and/or lack empathy, which enables them to purely focus on their own pleasure at the expense of other people’s well-being—under the adage ‘my pleasure first’. The second line of evidence on the behavioural origin of sadism could imply that repeatedly engaging in antisocial behaviour can over time desensitize an aggressor, and ultimately even cause them to enjoy hurting others. This was also supported by a bug-grinding study in which participants were either instructed to initially grind one or five bugs; this study showed that greater initial killing led to more favourable affective changes [23••].

### Differential Status of Sadism and Other Dark Triad Traits

Both clinical and community studies show a strong and consistent link between sadism and other Dark Triad traits. Our review shows that sadism is most strongly linked to psychopathy. One recent study examined whether these two traits can be empirically differentiated in the first place and had large community samples complete multiple questionnaires on the subcomponents of sadism and psychopathy [55]. The authors compared different factor models and found virtually identical fits of one- and two-factor models. In addition, both sadism and psychopathy were similarly correlated with other constructs such as hostility, antagonism, impulsivity, and dominance striving (positive correlations), and empathy, agreeableness, and conscientiousness (negative correlations). This caused the authors to defend a one-factor model based on the principle of parsimony. Other models that highlight the communalities between sadism and other Dark Triad traits are that of the *Dark Tetrad*—in which subclinical sadism has been added to the Dark Triad [33, 39•, 56]—and the *Dark Personality* [57•]. The latter model posits that an even wider range of dark traits aside from those of the classical tri-partite model, like egotism and spitefulness, including sadism, can all be boiled down to only one common core, called the Dark Factor (or shortly, *D*). Based on large-scale studies on the core and unique content of *D* and its predictive validity, the authors argue that *D* (as a single construct) is better suited to predict other maladaptive traits and behaviours than separate “dark” traits [57•].

That being said, other approaches continue to consider sadism as a unique trait that cannot be subsumed under one higher-order factor such as *D*. One study [41•], for example, found a superior fit for a bi-factor model that specifies a general antagonistic factor plus a specific sadism factor that reflects deriving pleasure from hurting others (see also [58]). This notion was further supported by data showing that sadism specifically predicts enjoyment of internet trolling, even when other Dark Triad traits are statistically controlled for [22, 37••]. Even studies eventually favouring the *D* factor approach suggest that, next to psychopathy, sadism is related to specific criteria after controlling for *D*, like self-centredness, insensitivity, and aggression [57•]. Put differently, while showing similar nomological networks, sadism comprises aspects not entirely reflected in *D*, and still remains theoretically distinct from psychopathy. Importantly, the overlap between sadism and other dark traits might also be partially attributable to the use of specific items that characterize multiple dark traits in measurement scales. Therefore, measures are needed that better differentiate between sadism and psychopathy, which can help dissolve the jingle-jangle fallacy of two differential concepts that often appear identical in analyses [59].

### Self-Report vs. Behavioural Measures

Sadism studies rely almost exclusively on self-report measures. This is understandable as self-report measures require fewer resources than, for instance, behavioural measures. Furthermore, people might feel more comfortable disclosing sadistic thoughts, fantasies, and behaviours in a self-report format. In addition, ethical and practical constraints make it difficult to study genuine interpersonal cruelty in the laboratory.

Nonetheless, striving for social desirability likely biases responses toward understating how much one enjoys hurting others or witnessing others’ pain [60, 61]. This poses a major threat to the validity of self-reported sadism. Some recent studies, in which participants had the opportunity to grind bugs or noise-blast an opponent, showed that mostly psychopathy’s coldheartedness component predicted enjoying bug grinding [47•, 48•]. While such behavioural approaches clearly reduce the chance of social desirability, self-report and behavioural sadism measures do not always align. In other words, people scoring high on sadism scales do not always behave like sadists. While such discrepancies potentially reflect differential manifestations of sadism (i.e. animal- versus human-directed, or dispositional versus state-level sadism), this line of research is only still in its infancy and deserving of further empirical attention.

### Moderating Factors

So far, the current review only discussed the existing correlational findings on the personality-sadism link. Importantly, these studies do not evidence that personality traits exert a causal impact on sadism. In addition, it is possible that the impact of personality on sadism is further enhanced (i.e. moderated) by contextual or individual-level factors.

One such individual-level factor is victim attitude, that is, the perpetrator’s evaluative feelings about the victim of their sadistic impulses. The subcomponent of victim attitude that has been studied most extensively is *perceived similarity*. Increasing incidental similarity between a participant and a confederate (e.g. by informing participants that they share their first name, birthday, or fingerprint) has been shown to decrease aggressive tendencies [62]. Martens and colleagues [23••] showed that participants who perceived themselves as more similar to bugs killed fewer bugs during a self-paced extermination task. Another type of victim attitude is *objectification*, where other people are evaluated mainly with regard to their “utility” for oneself, and thereby deprived of human attributes such as autonomy, agency, and subjectivity [63]. Objectification leads to the denial of moral status [64] and the perception of others as less competent [65]. Objectification is traditionally studied in a sexual context, where it implies reducing others to physical objects that exist mainly for one’s sexual pleasure [66]. One large-scale questionnaire study with community members showed that trait sadism is part of the nomological personality traits network of sexual objectification [67]. Another recent study took a broader view by extending objectification to the more general tendency to e.g. evaluate others on the base of their usefulness and contacting people only when they need something from them, and found this to relate to increased narcissistic traits [68]. It therefore seems plausible that some personality traits encompassing an excessive focus on one’s own pleasure contribute to sadism via objectification. These findings imply that a negative victim attitude likely engenders increased aggression and possibly sadistic pleasures. One previous study from our lab using a bug-grinding task investigated the impact of experimentally inducing a positive attitude, or a negative attitude toward bugs [47•]. Participants killed more bugs in the negative attitude condition than in the positive attitude condition, consistent with the view that holding a negative attitude toward victims increases violence toward them. It did not, however, affect the (self-reported) enjoyment of killing bugs. Put differently, inducing a negative attitude toward bugs led to more killing, but not to higher sadistic pleasure.

Finally, there is emerging evidence that boredom can augment sadism. Boredom is most commonly defined as “the aversive experience of wanting, but being unable, to engage in satisfying activity” [69, p. 483]. It is a highly aversive affective state, which motivates people to seek stimulating, arousing, or pleasurable activities [10]. Boredom proneness has been associated with increased levels of trait sadism [70], psychopathy [71, 72], and (covert) narcissism [72]. Furthermore, experimentally induced boredom predicted sadistic behaviour like grinding worms and destroying other participants’ payments, but only among those with high dispositional sadism [70]. Further evidence for the impact of situational factors on sadism comes from a public goods game study that focused on antisocial punishment (i.e. costly punishing cooperative individuals) [20]. Specifically, participants assigned to an intuitive thinking condition (i.e. prompted to “make decisions from the gut and follow your intuition”) showed higher levels of antisocial punishment than those instructed to deliberately consider pros and cons of punishment. This implies that—compared to a deliberate cost–benefit mental state of mind—sadism operates more on automatic, intuitive pathways. Overall, the studies evidence that the interplay between personality and factors such as victim attitudes, boredom, and state of mind increase the chance of sadism.

### Suggestions for Future Studies

The most apparent limitation of studies on the sadism-personality link is the near-exclusive reliance on self-report measures in community-based convenience samples. Consequently, most research is conducted with young, highly educated samples with low cultural heterogeneity—making it unclear how generalizable these findings are to the general public and to clinical samples. For instance, gender is unequally represented in the conducted studies, with clinical, forensic samples being predominately male [28••], and non-clinical sample being predominantly female. This can be problematic as both sadism and personality traits might manifest differently in men and women. Thus, replications in more educationally and culturally diverse and clinical samples are required. These studies should focus on a broader range of personality disorders, and include behavioural assessments of sadism and sadistic pleasure.

The current review finds the strongest evidence for a close relationship between sadism and psychopathy. This raises the question of how distinct both concepts are. To illuminate this more rigidly, it would be fruitful to further examine their independent contribution to, for instance, aggressive behaviour and criminal recidivism. Both sadism and psychopathy might also exert a combined augmented impact on hurting others.

Future studies would benefit from an increased focus on how sadism relates to the maladaptive personality traits formulated in DSM-5`s Alternative Model of Personality Disorders (AMPD) [16] and in the ICD-11 personality disorder chapter [25]. So far, there was only one study on the former that showed self-reported sadism traits to relate mostly to AMPD’s callousness facet within the antagonism trait [73]. It could also be expected that sadism would further relate to the AMPD facets of hostility, manipulativeness, impulsivity, or deceitfulness—but the fact that this was not the case could potentially be ascribed to the study sample consisting of older adults [73]. For the ICD-11, it could be expected that sadism would relate to dissociality and disinhibition traits.

Finally, further longitudinal studies are needed to assess which personality traits have a potentially causal influence on sadism in the sense that these traits can be regarded as risk factors for developing sadistic dispositions. In other words, future research needs to illuminate (a) the specific psychological processes underlying the causal effect of personality on sadism-related behaviour and (b) the conditions under which this effect is larger vs. weaker.

## Conclusion

Sadistic pleasure—the enjoyment of harm-infliction to others—has devastating interpersonal and societal consequences. Research on the link with DSM personality disorders is very scarce. Most studies focus either focus on Dark Triad traits in convenience samples, or on sexual sadism in forensic samples, and generally evidence that psychopathic and narcissistic traits relate to increased sadism. Being a topic in its infancy, much research is needed still to clarify the link between personality (disorders) and sadism. This includes studies in more diverse samples, behavioural assessments of sadistic pleasure, and studies on the interplay of such personality traits with situational, affective, and victim attitudes.

## Footnotes

1 *β*s reflect standardized regression coefficients, in multiple regression models where sadism was predicted by the three cluster PD scores.
